# REST upregulates gremlin to modulate diffuse intrinsic pontine glioma vasculature

**DOI:** 10.18632/oncotarget.23750

**Published:** 2017-12-28

**Authors:** Shavali Shaik, Bridget Kennis, Shinji Maegawa, Keri Schadler, Yang Yanwen, Keri Callegari, Rishi R. Lulla, Stewart Goldman, Javad Nazarian, Veena Rajaram, Jason Fangusaro, Vidya Gopalakrishnan

**Affiliations:** ^1^ Department of Pediatrics, University of Texas, MD Anderson Cancer Center, Houston, Texas, USA; ^2^ Department of Molecular and Cellular Oncology, University of Texas, MD Anderson Cancer Center, Houston, Texas, USA; ^3^ Department of Pediatrics, Northwestern Feinberg School of Medicine, Northwestern University, Chicago, Illinois, USA; ^4^ Department of Integrative Systems Biology, George Washington University School of Medicine and Health Sciences, Washington, District of Columbia, USA; ^5^ Department of Pathology, University of Texas Southwestern Medical Center, Dallas, Texas, USA; ^6^ Center for Cancer Epigenetics, University of Texas, MD Anderson Cancer Center, Houston, Texas, USA; ^7^ Brain Tumor Center, University of Texas, MD Anderson Cancer Center, Houston, Texas, USA

**Keywords:** DIPG, REST, vasculature, gremlin, VEGFR2

## Abstract

Diffuse intrinsic pontine glioma (DIPG) is a highly aggressive glial tumor that occurs in children. The extremely poor median and 5-year survival in children afflicted with DIPG highlights the need for novel biology-driven therapeutics. Here, we have implicated the chromatin remodeler and regulator of brain development called *RE1* Silencing Transcription Factor (REST), in DIPG pathology. We show that REST protein is aberrantly elevated in at least 21% of DIPG tumors compared to normal controls. Its knockdown in DIPG cell lines diminished cell growth and decreased their tumorigenicity in mouse intracranial models. DIPGs are vascularized tumors and interestingly, REST loss in DIPG cells also caused a substantial decline in tumor vasculature as measured by a decrease in CD31 and VEGFR2 staining. These observations were validated *in vitro*, where a significant decline in tube formation by human umbilical vein endothelial cells (HUVEC) was seen following REST-loss in DIPG cells. Mechanistically, REST controlled the secretion of a pro-angiogenic molecule and ligand for VEGFR2 called Gremlin-1 (GREM-1), and was associated with enhanced AKT activation. Importantly, the decline in tube formation caused by REST loss could be rescued by addition of recombinant GREM-1, which also caused AKT activation in HUVECs and human brain microvascular endothelial cells (HBMECs). In summary, our study is the first to demonstrate autocrine and paracrine functions for REST in DIPG development. It also provides the foundation for future investigations on anti-angiogenic therapies targeting GREM-1 in combination with drugs that target REST-associated chromatin remodeling activities.

## INTRODUCTION

Diffuse intrinsic pontine glioma (DIPG) is a highly aggressive glial tumor that occurs primarily in children between the ages of 5 and 10. DIPG originates in the pons, an area of the brain responsible for many essential functions such as coordination, breathing and heartbeat [1–3]. The median survival period for children with DIPG is currently only around 9 months, and the 5-year survival is less than 1% [4, 5]. The precarious location of the tumor precludes surgery [6–8]. It is also a highly infiltrative tumor and chemotherapy has modest efficacy at best. Therefore, focal radiation therapy is the current accepted standard of care [1, 9, 10]. Thus, there is a clear need for new therapies based on a better understanding of tumor biology. In the past, lack of DIPG tumor tissue and absence of animal models hampered investigations on tumor biology and therapeutics discovery. However, in recent years, availability of biopsy and access to tumor tissue samples resulted in successful development of DIPG cell cultures and high throughput genomic analyses of DIPG tumors. It has now become possible to pursue investigations at the molecular level [11–18].

DIPG are classified as Grade II-IV gliomas that include diffuse astrocytoma, anaplastic astrocytoma or glioblastoma. Under new WHO classification many DIPGs are defined as diffuse midline gliomas with H3K27M mutation [19]. The H3K27M mutation is a hallmark of more than 80% of DIPGs, implicating epigenetic perturbations in DIPG genesis [20]. The role of this mutation in tumor development is under active investigation in a number of groups [21–26]. Mutations in ACVR1 and TP53, amplification in PDGFαR and aberrations in MYC and MYCN are also found in a sub-set of DIPG patients [14, 27–29]. PI3K and MAPK signaling pathways are highly activated in a majority of DIPG tumors [30].

Here, we have focused on delineating the role of a chromatin remodeler called *RE1* Silencing Transcription Factor (REST) in DIPG development. REST is a zinc finger DNA binding protein and is associated with two independent chromatin-remodeling complexes at its amino (N-) and carboxy (C-) terminus [31–33]. It is regulator of brain development and most studies have focused on its function as a negative regulator of neuronal lineage specification in embryonic stem cells and neural progenitors [34–43]. REST expression is dysregulated in various tumors of neural or neural crest origin including medulloblastoma [44, 45], glioblastoma [46, 47], Ewings sarcoma [48, 49] and neuroblastoma [50–52]. Previous work from our group and others has shown that REST is important for medulloblastoma progression and maintenance [53]. However, REST biology in DIPG has not been evaluated thus far.

Here we show that REST gene and protein expression is elevated in DIPG samples compared to normal controls. It is also expressed to various levels in DIPG cell lines. REST loss diminished DIPG cell growth *in vitro* and formation of intracranial tumors. This was due to a decrease in cell proliferation. In addition, DIPG tumors resulting from cells with REST loss exhibited a decrease in CD31, an endothelial marker, and vascular endothelial growth factor receptor 2 (VEGFR2) staining. *In vitro* assays revealed a significant decrease in the ability of human umbilical vascular endothelial cells (HUVEC) to form tubes when cultured in medium harvested from DIPG cells where REST expression was knocked down. This change in tube formation was not due to endothelial cell proliferation. In mechanistic studies, we observed that levels of REST and that of the pro-angiogenic protein and ligand for VEGFR2, Gremlin-1 (GREM-1), were directly correlated in DIPG xenografts. REST knockdown caused a decline in secreted GREM-1 as measured by ELISA. Knockdown of *GREM-1* decreased the ability of DIPG cells to support the formation of tubes *in vitro* by both HUVEC and human brain micro-vascular endothelial cells (HBMECs). The ability of GREM-1 to promote downstream AKT activation in HUVEC and HBMECs was confirmed using recombinant GREM-1. Thus, our study is the first to implicate REST in DIPG tumors. We also demonstrate an autocrine and paracrine function for REST in DIPG development. The latter involves upregulation of GREM-1 and AKT activation.

## RESULTS

### REST is expressed at variable levels in human DIPG

**Figure 1:**
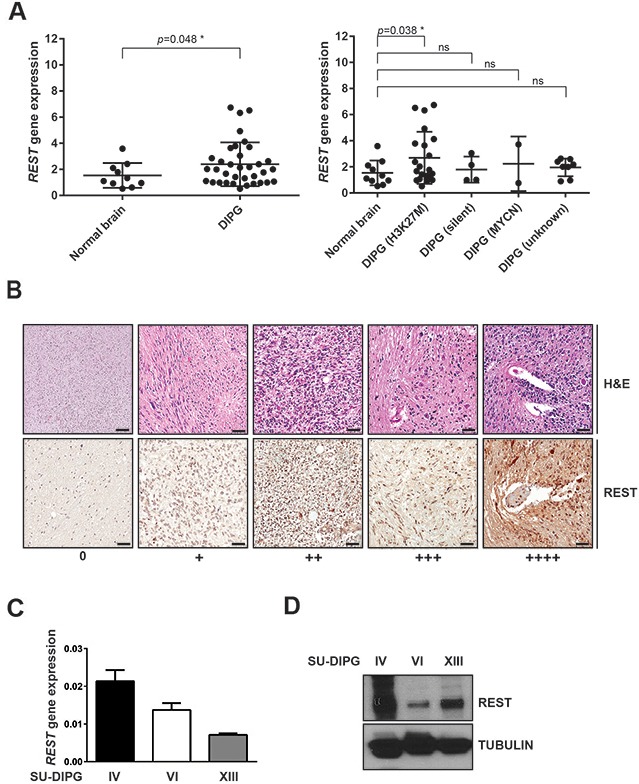
REST expression is elevated in human DIPG **(A)** Gene expression profiles measured by microarray. Gene expression datasets deposited in GEO were retrieved and analyzed using GEO2R as described in Materials and Methods. A comparison between normal brain samples and a total of 35 DIPG patient samples were shown on the left side. The same set of patient samples were subdivided into three distinct subgroups (H3-K27M, silent and MYCN) [16] and were compared with samples of an unknown subgroup on the right side. Each dot corresponds to one individual patient. Bars represent mean with standard deviations. ^*^p<0.05; ns=non-significant. **(B)** Hematoxylin-eosin (H&E) and immunohistochemical analysis (IHC) for REST in FFPE DIPG tumor specimens (n=19) and normal pons (n=2) was performed as described in materials and methods. Staining was scored by a neuropathologist as negative (0), weak and focal (+1), weak diffuse or multifocal (+2), strong and focal (+3), strong diffuse or multifocal (+4). Scale bar, 50μm. *REST* gene expression and protein levels in SU-DIPG-IV, -VI and -XIII cell lines were determined by **(C)** Q-RT-PCR and **(D)** Western blotting respectively. *GAPDH* was to normalize REST gene expression. Tubulin served as a loading control for Western blot analysis.

To evaluate REST expression in DIPG, we obtained microarray datasets containing gene expression values in human DIPG tumors from Gene Expression Omnibus (www.ncbi.nlm.nih.gov/geo) and analyzed through the GEO2R interface. REST mRNA levels were significantly elevated in DIPG tumor samples (n=35) compared to normal brain (n=10). This elevation was particularly significant in DIPGs with H3K27M mutation (Figure 1A). Further, human formalin-fixed paraffin-embedded (FFPE) DIPG specimens (n=19) obtained at autopsy were subjected to immunohistochemical (IHC) analyses. REST expression was scored by a neuropathologist as a negative (0)/ weak and focal (+)/ weak, diffuse or multifocal (++)/ strong and focal (+++)/or strong, diffuse or multifocal (++++). Normal brainstem samples are from patients with DIPG tumors, but from a region where tumor was thought not to be present. Approximately, 21% of tumors showed increased REST expression compared to total number of samples analyzed (Figure 1B; Table 1). REST transcript and protein levels in three human DIPG (SU) cell lines were determined by q-RT-PCR and western blotting. As shown in Figure 1C, REST mRNA levels were higher in SU-DIPG-IV and SU-DIPG-VI compared to SU-DIPG-XIII. However, REST protein levels were higher in SU-DIPG-IV and SU-DIPG-XIII relative to SU-DIPG-VI (Figure 1D).

**Table 1:** Immunohistochemical analysis of REST protein expression in FFPE human DIPG specimens

Sample	Highest Quantified REST staining Levels				
	0	+	++	+++	++++
Normal Brain Stem (2)			1	1	
Tumor (19)	2	4	4	5	4

REST expression was scored by a neuropathologist as a negative (0)/ weak and focal (+)/ weak, diffuse or multifocal (++)/ strong and focal (+++)/or strong, diffuse or multifocal (++++). Normal brainstem samples are from brainstems that had DIPG tumors but were read where tumor was not present.

### REST loss blocks DIPG cell growth *in vitro* and *in vivo*

**Figure 2:**
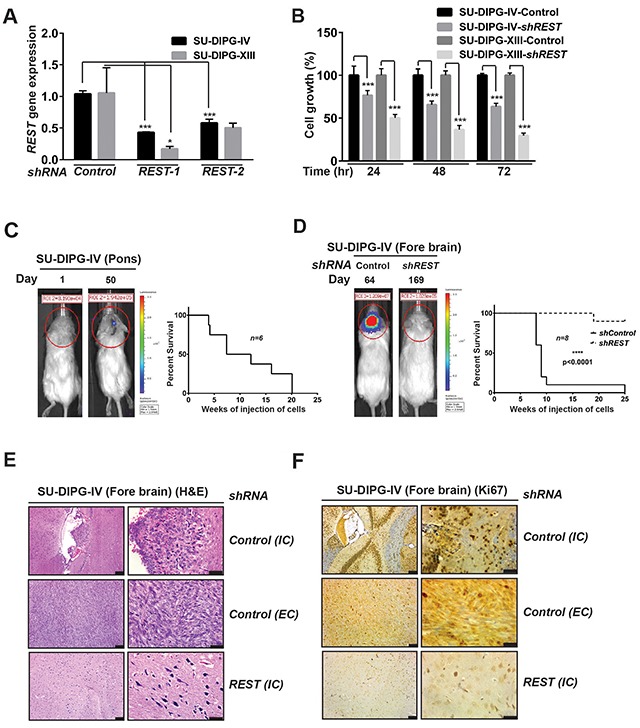
REST is required for DIPG growth **(A)** Efficiency of REST knockdown following lentiviral transduction of SU-DIPG-IV and –XIII cells using *shRNA* against *REST* or control *shRNA* was determined by Q-RT-PCR analyses. Significance was found to be ^***^p<0.001 (SU-DIPG-IV) and ^*^<0.05 (SU-DIPG-XIII). **(B)** DIPG growth was assessed using MTT assay. SU-DIPG-IV and SU-DIPG-XIII cells transduced with control *shRNA* or *shREST* were grown for 24, 48, 72 h, respectively and cell viability was determined by MTT assay at the indicated time points. Significance was found to be ^***^p<0.001 (SU-DIPG-IV), ^***^<0.001 (SU-DIPG-XIII). **(C)** Growth of SU-DIPG-IV- ffluc cells in the pons of NSG mice on day 1 and day 50 after implantation was assessed by bioluminescence imaging after injecting SU-DIPG-IV cells into the pons (n=6) (left panel). A Kaplan Meier survival curve is shown in the right panel. **(D)** Growth of SU-DIPG-IV-ffluc cells transduced with either control *shRNA* or *REST shRNA* into the forebrain of mice (n=8) was monitored by bioluminescence imaging (left panel). Kaplan Meier curves were generated to demonstrate a significant (p<0.0001) difference in survival between the 2 cohorts of mice. **(E)** H&E staining was performed to show the presence of robust intracranial (IC) and extracranial (EC) tumors in mice receiving injections of SU-DIPG-IV-ffluc cells transduced with control *shRNA* and sparser IC tumors in mice implanted with SU-DIPG-IV-ffluc cells transduced with *REST shRNA*. **(F)** IHC to measure Ki67 staining in tumors described in E. Scale bars in E & F, 50μm (left panel); 20μm (right panel).

To investigate if REST played a role in DIPG genesis, SU-DIPG-IV and SU-DIPG-XIII were transduced with lentivirus expressing a *control shRNA* or two different *shRNA* against *REST (shRNA1 and shRNA 2)*. REST expression was evaluated by Q-RT-PCR and was found to be knocked down by 60% in SU-DIPG-IV and 85% in SU-DIPG-XIII cell lines with *REST shRNA1* (Figure 2A). Similarly, 45% and 53% knockdown efficiencies were noted with both cell lines, respectively for *REST shRNA2* (Figure 2A). MTT assays were then performed to study the consequence of REST loss on the growth of SU-DIPG-IV and –XIII cells, *in vitro*. Growth was measured at 24, 48 and 72-hours post-transduction with control or *REST*-specific *shRNA*. As shown in Figure 2B, REST loss decreased cell growth relative to control shRNA expressing cells. This ranged between 76%-63% and 50%-29% for SU-DIPG-IV and -XIII cells, respectively (Figure 2B). These observations suggest that REST is required to sustain DIPG cell growth *in vitro*.

The requirement for REST in maintaining DIPG growth *in vivo* was examined in a mouse xenograft mouse model of DIPG. First, the ability of SU-DIPG-IV cells to grow in the pons of immunodeficient NOD scid gamma (NSG) mice was examined by stereotactic implantation of stably expressing firefly luciferase (ffluc) cells in mouse pons. Tumor growth was monitored by bioluminescence imaging (BLI) once a week. As shown in the representative image in Figure 2C (left panel), BLI signal was detected 50 days after orthotopic implantation of SU-DIPG-IV cells. Kaplan Meier analysis showed that all mice (n=6) succumbed to tumors by 20 weeks (Figure 2C, right panel). The effect of REST loss on tumorigenesis was studied through injection of *REST*-*shRNA1* or control *shRNA*-expressing cells into the forebrain [54]. Whereas 100% of animals (n=8 each) receiving injections of *control shRNA* expressing SU-DIPG-IV cells died within 7-24 weeks, bioluminescent signal was not observed in animals that were implanted with cells expressing *REST-shRNA1* even after 169 days (Figure 2D, left panel, data not shown). However, one mouse in the latter group died as shown in Figure 2D (right panel). Gross examination of the brains revealed a larger tumor burden in mice implanted with *control shRNA* expressing SU-DIPG-IV cells compared to mice implanted with SU-DIPG-IV cells expressing *REST-shRNA1* (Supplementary Figure 1A). Notably, the former group showed both intracranial (IC) and extracranial (EC) tumors, whereas substantially smaller IC tumors resulted from implantation of cells expressing *REST-shRNA1* (Figure 2D). Hematoxylin-eosin (H&E) staining of the brain sections is shown in Figure 2E. Immunostaining of brain sections showed significant Ki67 positivity in EC and IC in control shRNA expressing tumors in contrast to tumors resulting from *REST-shRNA1-*transduced cells (Figure 2E-2F and Supplementary Figure 1B). These observations indicate that REST is required for DIPG growth *in vivo*.

### REST modulates vasculature in DIPG tumors *in vivo*, and tube formation *in vitro*

**Figure 3:**
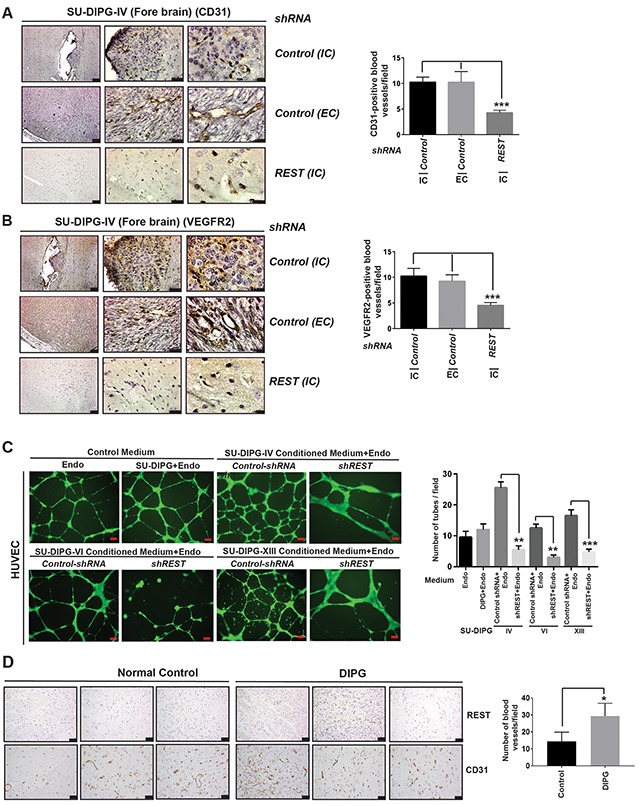
REST expression correlates with increase in number of blood vessels **(A)** Mice brains bearing tumors of SU-DIPG-IV cells expressing control *shRNA* (top and middle panel) or *shREST* (bottom panel) were stained with antibodies against the endothelial marker, CD31 (left panel). IC=intracranial tumors; EC=extra-cranial tumors. Quantitation of CD31-positive blood vessels between REST-expressing and REST-deficient DIPG tumors is shown in the right panel. Data shown is mean +/- SD, ^***^p<0.001, n=4. **(B)** The above brains were also stained with anti-VEGFR2 antibody (left panel) and quantification is provided in the right panel. Data shown is mean +/- SD, ^***^p<0.001; n=4. Scale bars in Figure A and B, 50μm (left panel); 20μm (middle panel) and 5 μm in right panel. **(C)** *In vitro* tube assay with human umbilical vein endothelial cells (HUVEC) were performed by culturing cells in endothelial medium alone or with unconditioned DIPG culture medium or endothelial medium with conditioned media from SU-DIPG-IV, -VI and -XIII cells stably transduced with either control *shRNA* or *REST-*specific *shRNA*. Endothelial medium and conditioned medium were used in a 1:1 ratio. Tube formation in matrigel was photographed after 16h (left panel) and quantitated (right panel). Data shown is mean+/- SD, ^**^p<0.01; ^***^p<0.001, n=4. Scale bar, 100μm. **(D)** Immunohistochemical analysis (IHC) for REST and CD31 in FFPE DIPG tumor specimens (n=3) and normal controls (n=3) was performed as described in materials and methods. Scale bar, 100μm. Data shown is for CD31 quantification, mean+/- SD, ^*^p<0.05 (right panel). CD31 positive blood vessels were counted in four different areas in each normal or tumor sample. An average number of vessels from 3 normal control and 3 DIPG tumor samples are shown.

Interestingly, H&E sections also seemed to suggest REST-dependent increases in tumor vasculature. To further investigate this, SU-DIPG-IV cells were transduced with lentivirus expressing *REST* *shRNA1* or control *shRNA* and equal number of viable cells were implanted into the forebrain of NSG mice (n=6). Tumors were allowed to grow and mice were sacrificed, their brains harvested and sectioned for IHC staining using CD31 antibody to mark endothelial cells. As seen in Figure 3A, a significant difference in CD31 staining and tumor vasculature was observed between *REST shRNA1* and control *shRNA* expressing intracranial (IC) and extracranial (EC) tumors. The number of vessels were quantitated to arrive at the plot shown in the right panel of Figure 3A (and Supplementary Figure 2A), wherein a significant decrease in the number of blood vessels was observed in *REST* *shRNA1-*expressing tumors compared to IC and EC tumors in mice implanted with cells expressing *control shRNA*. Levels of the pro-angiogenic receptor VEGFR2 were also found to parallel CD31 in these tumors (Figure 3B and Supplementary Figure 2B). These results suggested an involvement of REST-in modulation of DIPG vasculature.

*In vitro* assays were also performed to follow up on the above observations. Tube formation assay is a rapid and quantitative method to study the effects of compounds or conditioned media on formation of blood vessel like tubes in matrigel by endothelial cells [55]. To this end, human umbilical vein endothelial cells (HUVEC) were incubated with the conditioned medium obtained from SU-DIPG-IV, -VI and -XIII cells transduced with lentiviral expressing *REST* *shRNA1* or control *shRNA*. We observed that HUVEC incubated with conditioned medium from REST-expressing SU-DIPG-IV, -VI and -XIII cells formed significantly higher (2-3 fold) number of tubes in matrigel compared to HUVEC incubated with the medium from DIPG cells with REST expression knocked down (Figure 3C). An increase in HUVEC cell growth was not seen under our experimental conditions, indicating that the conditioned medium from REST-expressing DIPG cells was not due to an effect on HUVEC cell numbers (Supplementary Figure 2B). To establish a correlation between REST levels and vasculature, human DIPG FFPE samples and sections from non-tumor brain stems were subjected to IHC using anti-REST and anti-CD31 antibodies. Two out of three tumor samples were positive for REST staining, whereas REST was absent in normal brain stem samples. Furthermore, a significant increase in the number of blood vessels, as measured by CD31 staining, was seen in DIPG specimens relative to normal controls (Figure 3D, right panel). These observations suggest that the increase in CD31 in DIPGs was tumor specific and dependent on REST, and was not due to normal variations in vasculature in the brain stem.

### REST increases GREM-1 secretion

**Figure 4:**
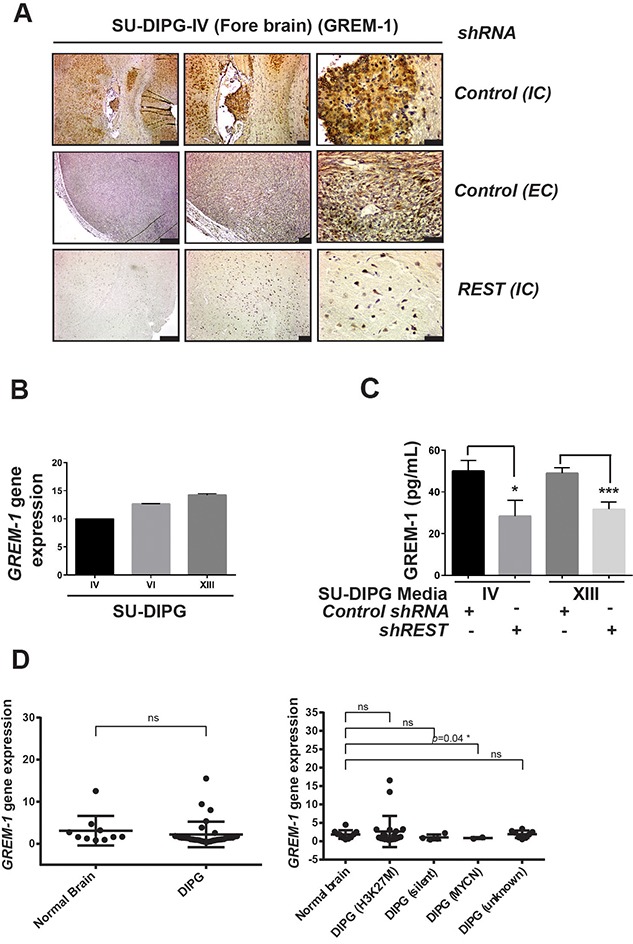
GREM-1 is secreted by DIPG cells in a REST-dependent manner **(A)** Tumor-bearing brains of mice implanted with SU-DIPG-IV cells stably expressing either control *shRNA* or *REST-*specific *shRNA* were analyzed by IHC for GREM-1 expression. IC=intracranial tumors; EC=extracranial tumors. Scale bars, 200μm (left panel); 50μm (middle panel) and 20 μm in right panel **(B)** *GREM-1* gene expression in SU-DIPG -IV, -VI and –XIII cells were obtained by Q-RT-PCR measurements. *GREM-1* expression was normalized to *GAPDH*. **(C)** GREM-1 secretion in conditioned medium from SU-DIPG (-IV and -XIII) cells transduced with either control *shRNA* or *REST-*specific *shRNA* was measured by ELISA. GREM-1 levels were expressed as pg/mL. Data shown as mean+/- SD, ^*^p<0.05; ^**^p<0.01; n=3. **(D)** Gene expression datasets deposited in GEO were retrieved and analyzed for *GREM-1* gene expression using GEO2R as described in Materials and Methods. A comparison between normal brain samples (n=10) and DIPG patient samples (n=35) were shown. Each dot corresponds to one individual patient. Bars represent mean with standard deviations. ^*^p<0.05; ns=non-significant.

Since VEGFR2 was found to be elevated in DIPG xenograft tumors *in vivo*, we focused on known ligands for this receptor. Vascular endothelial growth factor (VEGF) is a key ligand for VEGFR2 [56]. We first searched the microarray datasets containing gene expression values for VEGF expression in human DIPG and normal controls as explained in Materials and Methods section. As shown in Supplementary Figure 3A-3C, expression of VEGF isoforms -A, -B and -C was not significantly different between normal controls and DIPG tumors as a group or when sub-grouped based on their H3K27M status. Analyses of conditioned culture medium from SU-DIPG-IV, -VI and –XIII cells using a commercially available human angiogenesis proteome profiler array kit did not reveal VEGF protein in the three cell lines (Supplementary Figure 3D). A second ligand that binds VEGFR2, and one which is known to promote angiogenesis is Gremlin-1 (GREM-1) [57–60]. IHC revealed that SU-DIPG-IV xenografts did indeed express GREM-1 at higher levels compare to tumors from REST-deficient isogenic cells (Figure 4A). *GREM-1* gene expression was also detected by Q-RT-PCR in all 3 DIPG lines (Figure 4B), supporting GREM-1 as a candidate downstream effector of REST-mediated pro-angiogenic effect on HUVEC. REST-dependent changes in GREM-1 levels were also measured by ELISA, using conditioned medium from REST-expressing or REST-deficient SU-DIPG-IV cells. A significantly higher level of GREM-1 was detected in media from DIPG cells transduced with control *shRNA* compared to medium from *shREST* DIPG cells (Figure 4C). Interestingly, a REST-dependent difference in *GREM-1* gene expression was not observed in DIPG patient samples when compared to normal controls, with the exception of a small decrease in DIPG MYCN subgroup with a very limited number of specimens (Figure 4D). These findings suggest that REST may control GREM-1 at the post-transcriptional level.

### GREM-1 promotes tube formation and AKT activation in HUVEC and HBMEC

**Figure 5:**
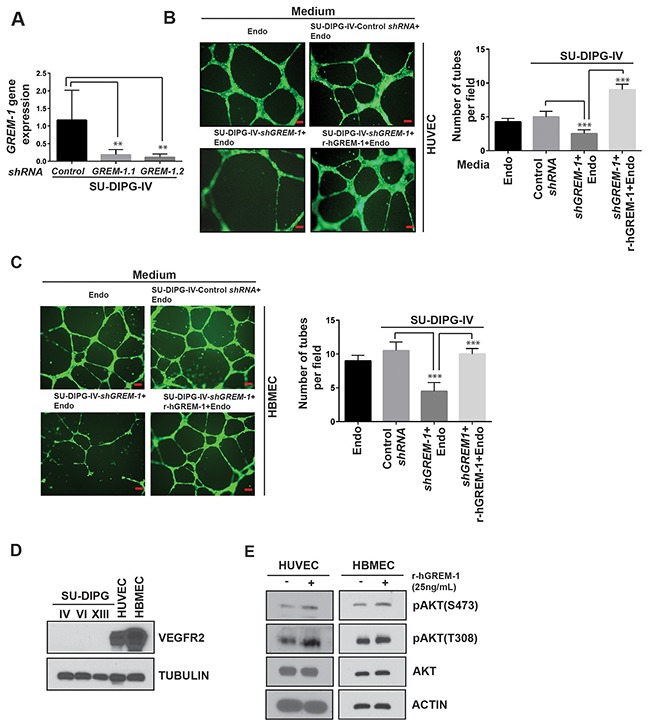
GREM-1 is required for tube formation *in vitro* **(A)** Q-RT-PCR analysis of *GREM-1* gene expression in SU-DIPG-IV cells stably expressing either control *shRNA* or *GREM-1*-specific *shRNA*. Lentiviral constructs expressing two different *GREM-1 shRNAs* (*GREM-1.1 and GREM-1.2*) were used to knockdown *GREM-1*. Efficiency of *GREM-1* knockdown was determined by Q-RT-PCR and expression was normalized to *18s* RNA. Significance is as shown (^**^p<0.01) **(B-C)** HUVEC or human brain microvascular endothelial cells (HBMEC) were cultured in endothelial cell medium and or conditioned medium from either control *shRNA* or *shGREM-1.2* transfected SU-DIPG-IV cells. Tube formation in matrigel was measured after 16h and images were obtained. The ability of GREM-1 to rescue loss of tube formation upon REST knockdown was determined by addition of human-recombinant GREM-1 (rGREM-1) to conditioned media-endothelial media mix. Scale bars, 100μm. (C) Quantification of tubes in matrigel shown in Figure B (right panels). Data shown is mean +/- SD, ^***^p<0.001, n=3. **(D)** Western blot analysis to assess VEGFR2 levels in SU-DIPG-IV, -VI and –XIII cells, HUVECs and HBMECs was done using anti-VEGFR2 antibodies. Tubulin served as a loading control. **(E)** Western blot analysis was performed to assess AKT signaling downstream of GREM-1 interaction with its potential receptor VEGFR2 in HUVEC and HBMEC. Anti-pAKT (S473), anti-pAKT (T308), total AKT, and anti-actin were employed.

**Figure 6:**
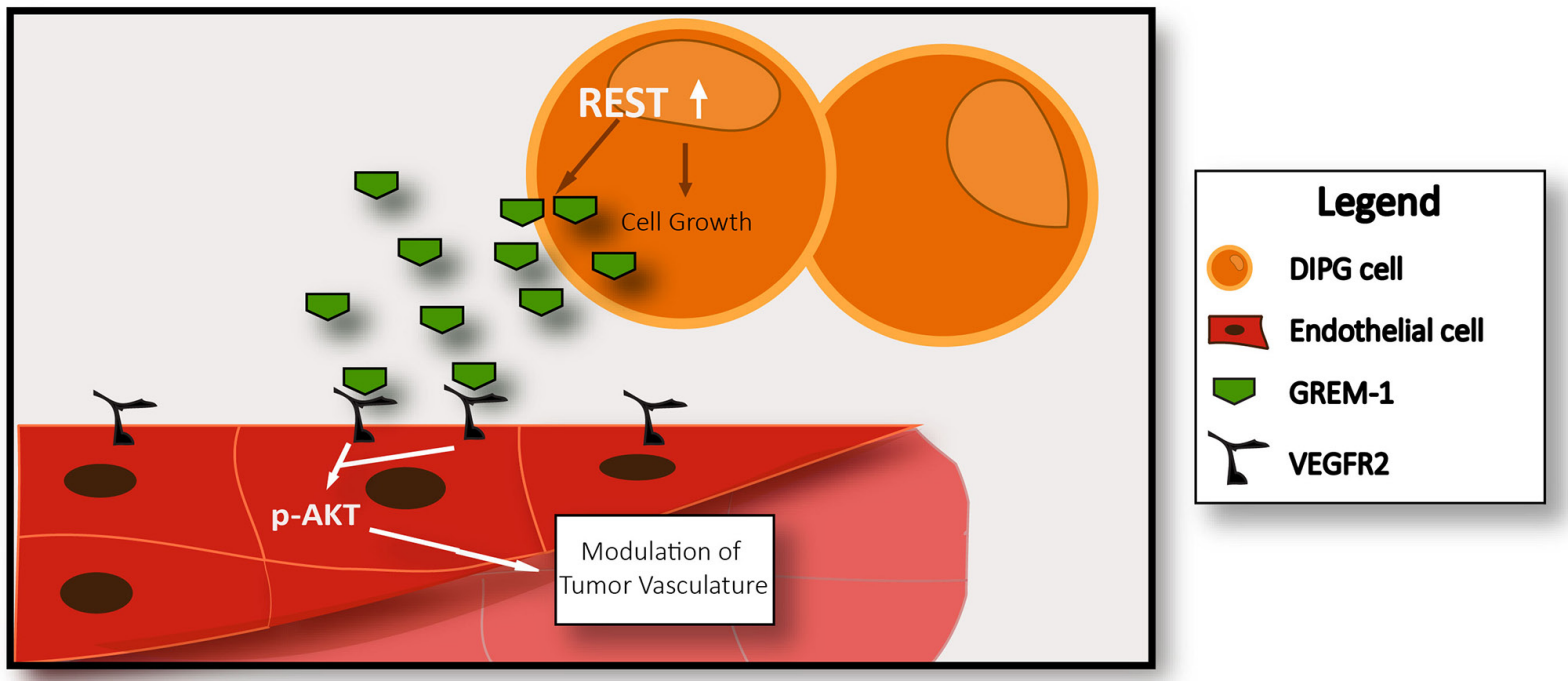
Model to explain mechanism of REST-mediated control of DIPG vasculature A pro-angiogenic molecule Gremlin is secreted by DIPG in a REST-dependent manner. Gremlin interacts with cell surface receptor VEGFR2 present in endothelial cells and causes increased vasculature. In addition, REST also controls DIPG growth.

To confirm the effects of GREM-1 on endothelial cells, the gene was knocked down in SU-DIPG-IV cells lentiviral transduction of two different *shRNAs* against *GREM-1* (*GREM-1.1* and *GREM-1.2*). Cells transduced with control *shRNA* were included for comparison. Efficiency of *GREM-1* knockdown was measured by Q-RT-PCR analysis and found to be approximately 85-90% (Figure 5A). As shown in Figure 5B, *GREM-1* loss in DIPG cells led to a significant decline in tube formation by HUVEC and HBMEC when compared to that supported by DIPG cells expressing GREM-1 (Figure 5B and 5C). Conversely, addition of recombinant human GREM-1 to conditioned medium from *shGREM-1.1* expressing DIPG cells restored tube formation activity of HUVEC and HBMEC (Figure 5B and 5C). As a first step, VEGFR2 protein expression was detected in HUVEC and HBMEC but not in SU-DIPG-IV, -VI and –XIII cells, as measured by Western blotting (Figure 5D). A downstream effect of GREM-1 interaction with its receptor VEGFR2 is activation of AKT signaling [61]. This suggested a paracrine effect of GREM-1 on HUVEC and HBMEC (Figure 5E). Together these findings suggest that REST elevates GREM-1 expression, which influences tube formation by endothelial cells in a paracrine manner (Figure 6).

## DISCUSSION

REST is a regulator of neuronal differentiation genes and has been mostly investigated in this context during normal brain development and brain tumors [31, 38, 40, 41, 45, 51, 53]. A recent study also demonstrated a novel role for REST in maintenance of genomic integrity during S phase in normal neural progenitors [33]. Consistent with these observations, REST appears to regulate brain tumor cell proliferation and tumor growth [62–65]. Other reports have implicated REST in the control of glioblastoma cell migration [66]. Aberrant REST expression has been associated with poor outcomes in several neural cancers, likely in part due to poor therapeutic response [50, 67]. These studies have largely focused on cell intrinsic activities influenced by REST. Here, we report both a cell-intrinsic and a cell-extrinsic contribution of REST towards the development of the pediatric brain tumor, DIPG. We show for the first time that a significant number of human DIPGs had elevated REST gene expression, particularly so in the H3K27M-positive samples. Additionally, 20% of human DIPGs express REST protein at elevated levels compared to normal controls. Although control tissues were obtained from “normal” areas distal to the tumor, DIPG is a diffusively infiltrative tumor and there certainly existed a possibility that stronger REST expression seen in our normal controls may be from invasive tumor cells. In effect, we may have underestimated the number of tumor samples with REST protein elevation. That this may indeed be the case is suggested by our observation that REST analyses of tumor samples we found that REST expression is extremely low to absent in control brain stem sections obtained from patients that did not have a brain tumor. Consistent with data from other neural tumors such as medulloblastoma, neuroblastoma and glioblastoma multiforme, REST is also required for maintenance of DIPG growth *in vitro* and *in vivo* [31, 34, 50]. Although, mechanisms remain to be uncovered, REST is known to control the expression of molecules involved in regulating cell proliferation such as p27 and USP37 [62, 63]. Another important area of investigation that needs to be followed up in the future centers around the possibility that REST activity in concert with histone H3K27 methylation may modulate the expression of target genes involved in development and lineage specification. Support for the above comes from published data which have shown an interaction between REST and polycomb repressive complex-2 (PRC2) [68, 69]. For example, co-recruitment of REST-co-REST-LSD1 and PRC2 to regulatory regions of bivalent genes through the long-noncoding RNA, *HOTAIR*, is thought to promote gene repression [68]. Yet other groups have shown a mutually exclusive occupancy of REST and PRC2 at consensus REST binding sites, known as *RE1* motifs, located near CpG islands of genes [69]. Here, REST expression would prevent PRC2 occupancy leading to a reduction in histone H3K27 trimethylation and consequent gene activation, whereas its loss would promote PRC2 binding, an increase in histone H3K27 trimethylation and silencing of target gene expression. Whether this relates to the variable correlation between DNA methylation status and histone H3K27 trimethylation at some genes but not others in H3K27M mutated DIPG cells, remains to be examined [70, 71].

Of additional interest is our finding that REST enhances DIPG vasculature. Magnetic resonance imaging (MRI) studies in DIPGs have shown that the average cerebral blood volumes in areas of occult enhancement are significantly higher than non-enhancing areas of the same tumor, raising the possibility that tumor development is accompanied by an increase in angiogenesis [72]. This study is the first to formally validate this observation in animal models of DIPG. It is also consistent with a previously described role for REST in modulating the vasculature in Ewing's sarcoma [49].

Angiogenesis, a process of sprouting of blood vessels from existing ones, is determined by a balance between pro-angiogenic factors and angiogenesis inhibitors [73–75]. Members of the vascular endothelial growth factor (VEGF) and fibroblast growth factor (FGF) family are well-studied inducers of angiogenesis [76–80]. Surprisingly, there were no significant differences in VEGF *mRNA* between normal controls and DIPG, and VEGF-C protein was not secreted by DIPG cells. However, GREM-1, a glycoprotein and member of the Dan family of cysteine knot-secreted proteins was found to be secreted by the three DIPG cell lines studied [81]. GREM-1 is a strong antagonist of signaling through bone morphogenic protein (BMP), a pathway that is believed to play an important role in DIPG development [82]. Although a number of studies on GREM-1 have examined its function in the control of patterning during embryonic development and regulation of early limb, lung, kidney and bone development, increased GREM-1 expression has also been described in various cancers including lung, kidney, ovary, breast, colon and pancreatic cancers [83–86]. GREM-1 expression in the normal neuronal and astrocytic compartments and its interaction with SLIT, a family of axonal guidance proteins, has been shown to control chemotaxis of monocytes in the brain [87, 88]. In glial-tumors such as GBM, GREM-1 overexpression in the cancer stem cells (CSCs) compartment was found to be necessary to maintain tumor cell growth *in vitro* and *in vivo* [89, 90]. *GREM-1* overexpression in glioma cell lines also promoted epithelial to mesenchymal transition (EMT) through upregulated expression of E-Cadherin and BMP7, and activation of TGF-β signaling through engagement of BMP receptors I and II [89]. Of specific relevance to observations in our study is the fact that GREM-1 is an agonist of vascular endothelial growth factor receptor, VEGFR2 [57]. Its interaction with VEGFR2 is known to control angiogenesis by promoting migration of endothelial cells [81]. This is consistent with our findings that ectopic GREM-1 expression does not affect endothelial cell growth, but does enhance tube formation *in vitro*. Expression of VEGFR2 in HUVEC and HBMEC *in vitro* and in the DIPG tumor vasculature *in vivo* also supports the possibility that GREM-1 acting through VEGFR2 may modulate DIPG angiogenesis (Figure 6). However, we cannot rule out additional roles for GREM-1 in DIPG development such as promoting EMT or immunomodulation, which has been described in other tumors [88, 91].

The molecular mechanisms underlying REST-dependent upregulation of GREM-1 is a subject of ongoing investigation. REST is associated with a number of chromatin remodeling enzymes, whose activity can be inhibited by drugs that are currently in use in the clinic or are being investigated in pre-clinical studies [5, 92–94]. These agents may have applications in the treatment of REST-expressing DIPGs in the future. Finally, our results also suggest that the efficacy of VEGF inhibitors targeting VEGF alone against these tumors may be limited and potential trials considering their use to target DIPG vasculature may have need to be re-evaluated [95]. Prophylactic anti-Gremlin antibodies have shown promise in pulmonary arterial hypertension in mice, which may support its examination either alone or in combination studies in animal models of DIPG [96].

## MATERIALS AND METHODS

### Analyses of DIPG samples

Immunohistochemical analyses of normal and tumor samples were performed by Dr. Veena Rajaram following Institutional Review Board (IRB) approval. Paraffin-embedded brain sections were immunohistochemically stained by hematoxylin eosin (H&E), and REST protein expression was analyzed using anti-REST antibody (Cat# IHC-00141; Bethyl, Montgomery, TX; 1:100 dilution) followed by DAB staining (Vector laboratories, Burlingame, CA). Slides were evaluated for REST levels by neuropathologist utilizing a 5-point grading scale as described in Figure 1A.

### Cell culture

SU-DIPG cell lines (IV, VI and XIII) were grown under conditions previously described [5]. 293T cells were grown in DMEM medium in the absence of serum. Human Umbilical Vein Endothelial Cells (HUVEC; Cat# CC2519) and EGM-2 bullet kit (endothelial basal media and growth factors; Cat# CC3162) were purchased from Lonza (Allendale, NJ) and were cultured according to manufacturer's recommendations. Human Brain Microvascular Endothelial Cells (HBMEC; Cat# HEC02) and Endo-Growth medium were purchased from Neuromics (Edina, MN) and cultured in complete Endo-Growth medium (Cat# MED001) in combination with EGM-2 media (1:4 ratio).

### Lentivirus production and transduction

293T cells were transfected with lentiviral constructs expressing GFP and *shRNA*s against *REST* or *GREM-1* were purchased from M.D. Anderson Cancer Center (MDACC) Core facility (shRNA and ORF-eome Core) and co-transfected with plasmids pax2 and MD2 (for packaging) using OptiMEM®I and Lipofectamine® 2000 Reagent (Cat# 31985-062 and 11668-019, Thermo-Fisher Scientific, Waltham, MA) according to protocols from MDACC Core facility. Lentiviral particles were harvested 48 hours after transfection by filtering supernatant using a 70μm syringe filter and spinning filtered medium at 1,500 RPM for 5 minutes. Transduction of SU-DIPG cell lines was done by incubating cells with *shRNA*-expressing lentivirus for 72 hours, and then flow sorted for GFP positive DIPG cells at the MDACC Cell Sorting Core facility. Knockdown of *REST* or *GREM-1* was confirmed by Q-RT-PCR. For *in vivo* experiments, GFP positive *REST*-knockdown SU-DIPG-IV cells were transduced with firefly luciferase co-expressing Mkate lentivirus and sorted for mKate. These cells were injected into NSG mice to develop tumors. Nucleotide sequences for *shRNAs* purchased from the *shRNA* and ORF-eome Core at MDACC are as follows.

shREST-1: 5’-TTGAAGTTGCTTCTATCTG-3’;

shREST-2: 5’-TTTGAACTGTAAATATCTG-3’,

shGREM-1.1: 5’-AGATTCTTACTTGGCTTAA-3’;

shGREM-1.2: 5’-ACCATGATGGTCACACTCA-3’

Control shRNA: 5’-ATCTCGCTTGGGCGAGA GT-3’.

### Q-RT-PCR

RNA was isolated from cells using Zymo Research Quick-RNA^TM^ MiniPrep Kit (Cat# 11-328; Genesee Scientific, San Diego, CA 11-328) and resuspended in water. RNA was quantified using NanoDrop 1000 (Thermos Fisher Scientific, Wilmington, DE). At least 300ng of RNA from each sample was reverse-transcribed using the Bio-Rad iScript^TM^ cDNA Synthesis Kit (Cat# 170-8891; Bio-Rad, Hercules, CA). Reverse transcription was completed on the Bio-Rad T100 Thermal Cycler (Bio-Rad, Hercules, CA). qPCR was performed on the Roche LightCycler® 96 using Roche LightCycler® 480 SYBR Green I Master mix (Cat# 04707516001; Roche, Indianapolis, IN) and 0.5μL of 10mM each primer. Relative mRNA expression after normalizing to GAPDH or 18s was determined by the comparative 2-ΔΔCT method [97]. Primer sequences are as below.

**Table:** 

Primer Name	Primer Sequence
Human *REST*-Forward	5’-*GGCAGCTGCTGTGATTACCT*-3’
Human *REST*-Reverse	5’-*AGTTGTTATCCCCAACCGGC*-3’
Human *GREM-1*-Forward	5’-*GCCAGACAAGGCCCAGCACAATGAC*-3’
Human *GREM-1*-Reverse	5’-*GGTATTTGCGCTCCGTCACATG*-3’
Human *GAPDH*-Forward	5’-*CGCTCTCTGCTCCTCCTGTT*-3’
Human *GAPDH*-Reverse	5’-*CCATGGTGTCTGAGCGATGT*-3’
Human *18s*-Forward	5’-*CGGCGACGACCCATTCGAAC*-3’
Human *18s*-Reverse	5’-*GAATCGAACCCTGATTCCCCGTC*-3’

### MTT assay

SU-DIPG-IV and /or SU-DIPG-XIII cells were plated in a 96-well plate at a density of 6,000 cells/well in 100μL complete media. At various times (24, 48 or 72h) after lentiviral transduction with control *shRNA* or REST-*shRNA,* 50μL of media was decanted, and 25ng of 98% Thiazolyl Blue Tetrazolium Bromide (MTT) (Cat# M2128-1G; Sigma, St. Louis, MO) was added. Cells were incubated for 2-4 hours before 100 μl of DMSO was added to each well and triturated to uniformly suspend the MTT precipitate. Plates were read for absorbance (570/650 nm) with a SPECTRAmax®PLUS^384^ Microplate Spectrophotometer (Molecular Devices, Sunnyvale, CA).

### *In vivo* assays

All animal experiments and procedures were approved by the Institutional Animal Care and Use Committee (IACUC). SU-DIPG cells were injected into the pons or forebrain of 4-6 month old NOD *scid* gamma null (NSG; NOD.*Cg*-*Prkdc^scid^ Il2rg^tm1Wjl^*/SzJ JAX) mice (The Jackson Laboratory, Bar Harbor, ME) [54]. SU-DIPG-IV stably expressing firefly luciferase (ffluc) were suspended in sterile PBS prior to employing a sterile field for injection of either 1 million SU-DIPG-IV-ffluc into the pons (1mm posterior to lamboid suture, 1mm lateral to midline, 5mm below skull surface) or 500,000 SU-DIPG-IV-ffluc into the forebrain (1.5mm anterior to lamboid, 2.5mm right of midline, 2mm deep), using a Harvard Apparatus (Holliston, MA) and 26 guage gas tight Hamilton syringe. Mice were anesthetized using 2-4% inhalant isoflurane prior to the surgical procedure. Bioluminescent imaging of DIPG tumors was performed using the Caliper LifeSciences IVIS Spectrum IVIS200 *in vivo* imaging system (Hopkinton, MA). Mice were injected with 150mg/kg with D-Luciferin (Cat# E1605; Promega Corporation, Madison, WI) through an intraperitoneal (IP) route, and sacrificed upon onset of clinical signs or signs of solid tumors formed in the brain and grown through skull. Brains were collected and sectioned for IHC analysis.

### Immunohistochemistry

Mice brain tissues were fixed in 10% neutral buffered formalin and embedded in paraffin blocks. 4-μm-thick brain sections were cut and used for immunohistochemical (IHC) analysis. After deparafinization and rehydration, heat-mediated antigen retrieval and blocking, sections were incubated with primary antibodies to REST (Cat# IHC-00141; Bethyl, Montgomery, TX), CD31 (Cat# ab28364; Abcam, Cambridge, MA), VEGFR2 (Cat# 2479; Cell Signaling Technology, Danvers, MA), Ki67 (Cat# AB9260; Millipore, Billerica, MA), and Gremlin-1 (Cat# PA5-13123; Thermo-Fisher Scientific, Waltham, MA) at 4°C overnight. After washing, sections were incubated with secondary antibody conjugated to horse radish peroxidase (Cat# 111-035-003 and Cat# 115-035-003; Jackson ImmunoResearch Labs, West Grove, PA) for 2 h at room temperature. All incubations were performed under humidified conditions. After washing, slides were developed using 3,3′-diaminobenzidine substrate (DAB; Cat# Vector Laboratories, Burlingame, CA), counterstained with hematoxylin, dehydrated, mounted and visualized under a microscope (Nikon ECLIPSE E200; Melville, NY). Images were obtained using an Olympus SC100 camera (Waltham, MA) attached to the microscope. Images were processed using CellSens Entry microscopy imaging software (Olympus Life Sciences, Waltham, MA).

### *In vitro* angiogenesis assay

*In vitro* angiogenesis assay (tube formation assay) was performed as described previously [98]. Briefly, Matrigel (Cat# 354230; Thermo Fisher Scientific, Waltham, MA; 100 μl/well) was placed in 96 well sterile culture plates. HUVEC or HBMEC (5 × 10^4^ cells per 25μL) mixed in conditioned medium from DIPG or plain DIPG medium (1:1 ratio) were placed on top of the matrigel and incubated in a CO_2_ incubator at 37°C. After 16 h, cells were stained with Calcein-AM (Cat# C3100MP; Thermo Fisher Scientific, Waltham, MA) for 30 min, rinsed with the endothelial cell medium. Tube formation in matrigel was visualized under a fluorescence microscope (Nikon Eclipse T*i-E*, Melville, NY) and images were taken with a camera (Andor, Zyla, Concord, MA) attached to the microscope. Image analysis and quantification was done using NIS elements AR software (Nikon, Melville, NY).

### Western blot analysis

Cell lysates were prepared by lysing DIPG cells or HUVEC or HBMEC in EBC lysis buffer (50 mM Tris, pH 8.0, 120 mM NaCl, and 0.5% NP-40) supplemented with protease inhibitors (Thermo Fisher Scientific, Waltham, MA) and phosphatase inhibitor cocktail-2 (Sigma, St. Louis, MO). Lysates were clarified and protein concentration in supernatants was measured using Bio-Rad protein assay dye reagent (Bio-Rad Laboratories, Hercules, CA). Samples were then subjected to SDS-PAGE and Western blot analyses with the following primary antibodies: REST (Cat# 07579; Millipore, Billerica, MA), pAKT^Ser473^ (Cat# 9271, Cell Signaling Technology (CST), Danvers, MA) p-AKT^T308^, (Cat# 5106; CST, Danvers, MA), AKT (Cat# 9272; CST, Danvers, MA), VEGFR2 (Cat# 2479; CST, Danvers, MA) and Tubulin-HRP (Cat# ab40742; Abcam, Cambridge, MA). After washing and incubation with the corresponding HRP-conjugated secondary antibodies (Jackson Immuno Research, West Grove, PA), membranes were developed using SuperSignal (Cat# 34075; Cat#34087; Thermo-Scientific, Waltham, MA) followed by autoradiography.

### Human angiogenesis proteome profiler assay

Proteome profiler human angiogenesis array kit (Cat# ARY007, R&D Systems Minneapolis, MN) was used to measure expression of 55 angiogenesis related proteins including VEGF-C. Conditioned media from SU-DIPG (-IV, -VI and -XIII) cells were collected for the assay as per manufacture's conditions.

### ELISA

GREM-1 levels in DIPG conditioned medium were quantified using a ELISA kit according to manufacturer's recommendations (Neo BioLab, Woburn, MA).

### GEO2R analysis

Microarray datasets containing the gene expression values of diffuse intrinsic pontine glioma (DIPG) patients were obtained from Gene Expression Omnibus (www.ncbi.nlm.nih.gov/geo). We used GSE50025 dataset which contained Illumina HT-12 V4 BeadChip Array profiling of 35 DIPG samples and 10 normal brain controls [16] to evaluate gene expression. The data were analyzed through the GEO2R interface (http://www.ncbi.nlm.nih.gov/geo/geo2r/) as previously described [99]. Microarray data were normalized in Partek Genomics Suite v6.6 using per-probe median-centered quantile normalization. p-values for comparisons between sample groups were obtained using the unpaired *t*-test with Welch's correction.

### Statistical analysis

Experimental data reported was Mean ± SD of a minimum of 3 samples. A P value of < 0.05 was considered to be statistically significant. Student's *t* test was performed for significance between groups. GraphPad Prism version 6.01 for Windows (GraphPad Software, San Diego, CA) was used to generate graphs.

## SUPPLEMENTARY MATERIALS FIGURES



## Abbreviations

DIPG: Diffuse Intrinsic Pontine Glioma
REST: RE1 Silencing Transcription Factor
GREM-1: Gremlin 1
VEGFR2: Vascular Endothelial Growth Factor Receptor-2
HUVEC: Human Umbilical Vein Endothelial Cells
HBMEC: Human Brain Microvascular Endothelial Cells
BLI: Bioluminescence Imaging
ffluc: firefly luciferase
NSG: Nod Skid Gamma
IC: Intracranial
EC: Extracranial
FFPE: Formalin-Fixed, Paraffin-Embedded.
