# Supplementary material for: REST upregulates gremlin to modulate diffuse intrinsic pontine glioma vasculature
Source: Oncotarget. 2017 Dec 28;9(4):5233–50. doi: 10.18632/oncotarget.23750 (PMC5797046; doi:10.18632/oncotarget.23750)
Supplement: Supplementary file 1 [file oncotarget-09-5233-s001.pdf]

REST upregulates gremlin to modulate diffuse intrinsic pontine glioma vasculature

SUPPLEMENTARY MATERIALS

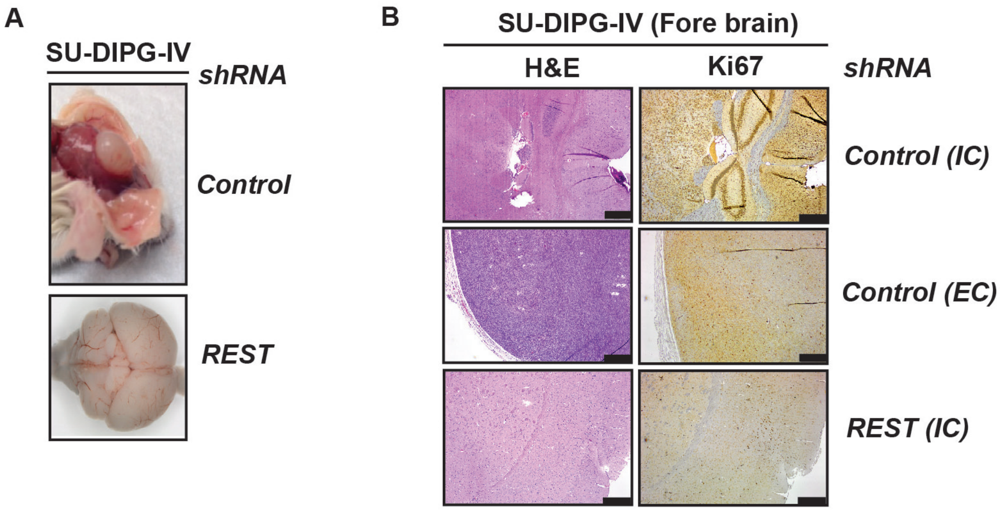

Supplementary Figure 1: REST promotes tumor growth *in vivo*. **(A)** Gross imaging was done to demonstrate a measurable difference in tumor burden in the forebrain area of mice injected with ffluc-tagged SU-DIPG-IV cells expressing control *shRNA* (upper panel) compared to animals implanted with isogenic cells expressing with *RESTshRNA* (lower panel). **(B)** Lower magnification (4X) images of H&E and Ki67 stained brain sections of mice injected with SU-DIPG-IV cells expressing control *shRNA* or *REST-shRNA*. Scale bars, 200µm (related to Figure 2).

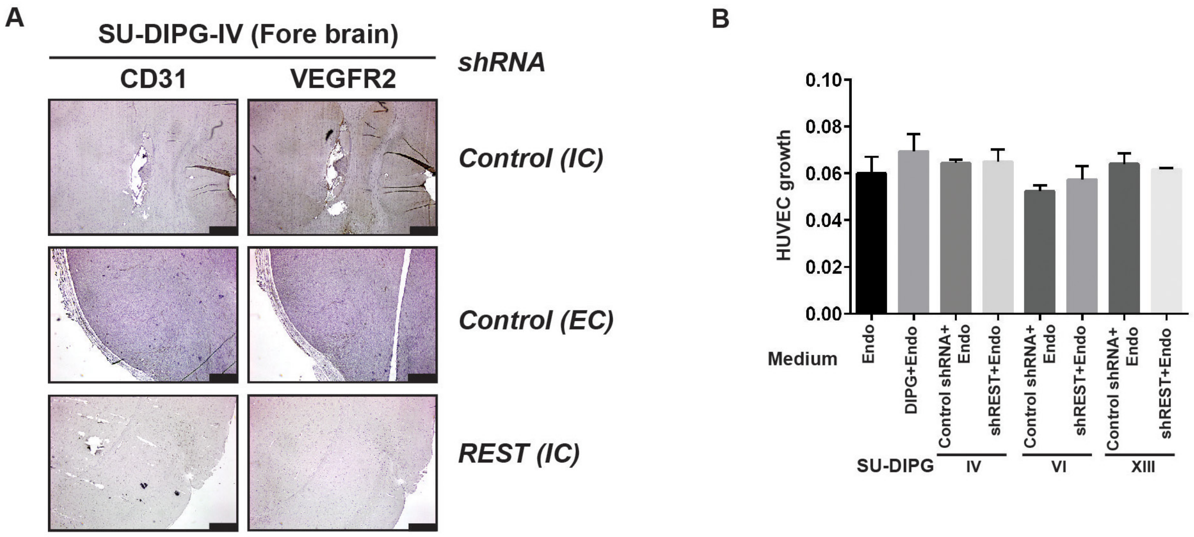

Supplementary Figure 2: REST expression promotes an increase in blood vessels in DIPG tumors. **(A)** Low magnification (4X) images ofIHCs for CD31 and VEGFR2 in tumor bearing mice brains injected with SU-DIPG-IV cells expressing control *shRNA* or *REST-shRNA*. Scale bars, 200µm (related to Figure 3). **(B)** MTT assays were performed to evaluate growth of HUVEC when cultivated for 16 h with conditioned medium from DIPG cells transduced with either control *shRNA* or *REST*-specific *shRNA*.

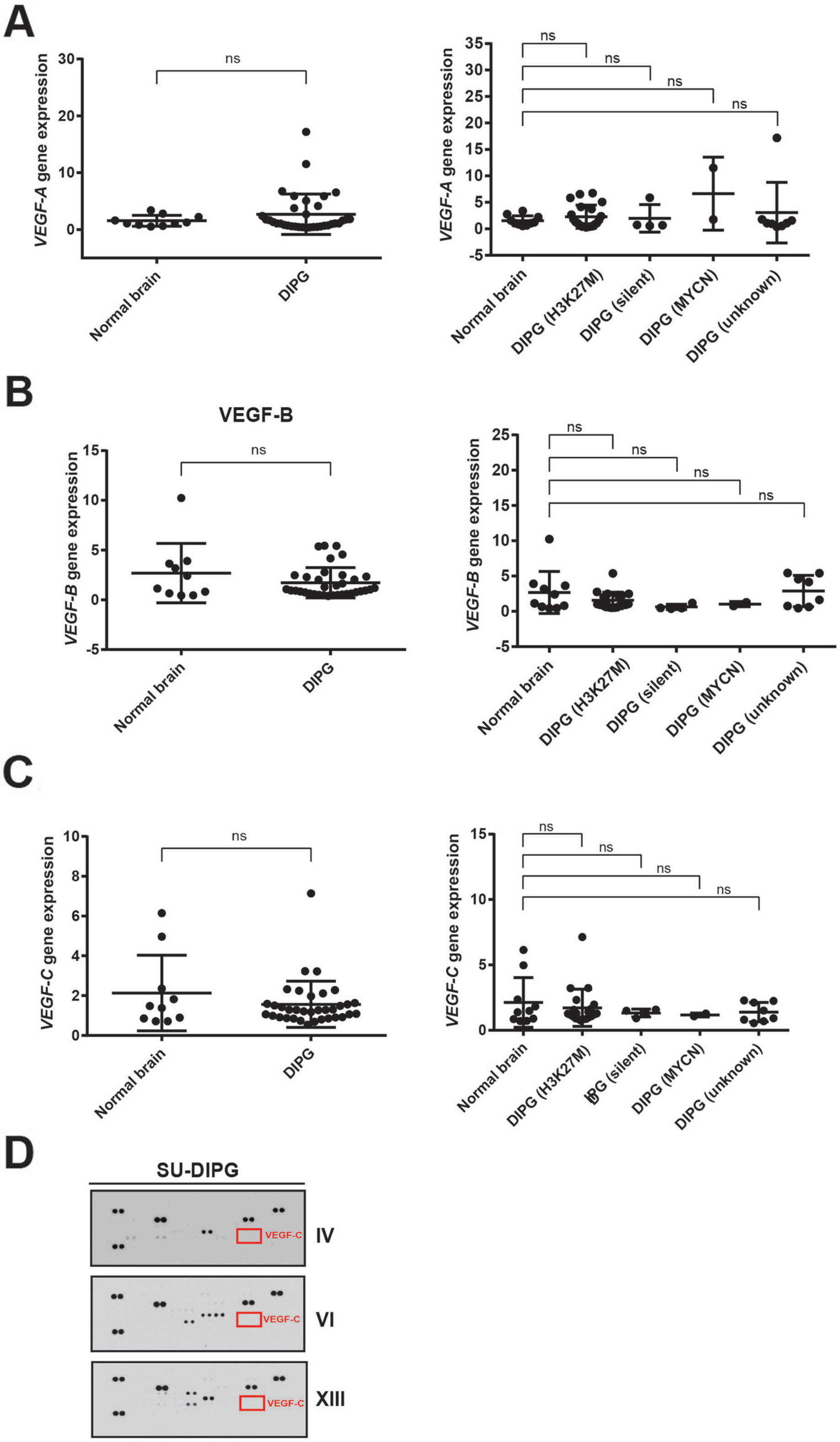

Supplementary Figure 3: VEGF profiles in DIPG. (A-C) Gene expression datasets deposited in GEO were retrieved and analyzed for VEGF-A, VEGF-B, VEGF-C *mRNA* expression using GEO2R. A comparison between normal brain samples (n=10) and DIPG patient samples (n=35) were shown. Each dot corresponds to an individual patient. Bars represent mean with standard deviations. **(D)** A commercially available human angiogenesis proteome profiler array kit was used to identify VEGF secreted by SU-DIPG cell lines -IV, -VI and -XIII) and demonstrate absence of VEGF in the conditioned medium of the above cells. (related to Figure 4).
